# Sequential antibody induction for immune tolerance in clinical organ transplantation: a feasibility review of immunosuppressant withdrawal protocols

**DOI:** 10.3389/fimmu.2026.1900794

**Published:** 2026-08-26

**Authors:** Yachao Li, Fuxia Chen, Xia He, Lian Yan, Anzhu Bao

**Affiliations:** 1 College of Medicine and Life Sciences, Chengdu University of Traditional Chinese Medicine, Chengdu, China; 2 Operating Room, Sichuan Provincial People’s Hospital, Chengdu, Sichuan, China; 3 Department of Laboratory Medicine, Ya’an People’s Hospital, Ya’an, China

**Keywords:** immune tolerance induction, immunosuppressants, organ transplantation, regulatory T cells, sequential antibody induction

## Abstract

Long-term immunosuppressant use after solid organ transplantation causes serious complications, including chronic rejection, infection, malignancy, and metabolic disorders, which has motivated the clinical pursuit of immune tolerance induction. Sequential antibody induction protocols – using peri-transplant antibodies (e.g., alemtuzumab, anti-thymocyte globulin, belatacept, anti-CD40 monoclonal antibodies) combined with phased reduction or withdrawal of maintenance drugs – aim to establish “operational tolerance” or complete tolerance. This review systematically examines the immunological rationale and major clinical strategies (T-cell depletion, costimulation blockade, mixed chimerism, and regulatory cell therapy). Based on efficacy, safety, and feasibility, we propose four novel sequential regimens with explicit evidence levels. Our multidimensional feasibility assessment – covering cost, complexity, risk–benefit, patient selection, and regulatory barriers – indicates that costimulation-blockade protocols currently offer the best balance for near-term clinical use, whereas chimerism approaches are the most effective but remain restricted to specialised centres. Future work should integrate precise immune stratification, advanced antibody engineering, and cell-based therapies to facilitate individualized sequential protocols, ultimately moving the field from lifelong immunosuppression to controlled immune tolerance.

## Introduction

Over the past 50 years, calcineurin inhibitors (CNIs) have raised 1-year graft survival after solid organ transplantation to over 90% (1). According to the US Organ Procurement and Transplantation Network (OPTN) annual report, 1-year graft survival rates for first-time kidney, liver, and heart recipients are 94.2%, 91.5%, and 91.0%, respectively (2). Long-term graft survival, however, has not improved at the same pace. For living-donor kidney transplants, 10-year graft survival is roughly 55–60%; for deceased-donor kidneys, it is only 45–50% (3). Chronic antibody-mediated rejection (AMR) and CNI nephrotoxicity are the main causes of late graft loss, together accounting for 60–70% of failures (3). The main causes of graft loss are chronic antibody-mediated rejection (AMR) and CNI nephrotoxicity, which together account for about 60–70% of late graft failures (3). In liver transplantation, although immunosuppression can be less intensive, long-term CNI exposure still leads to significant renal dysfunction in about 20–30% of recipients within 10 years, with cumulative incidences of new-onset diabetes and hypertension reaching 30% and 50%, respectively (4).

Lifelong immunosuppression also exposes recipients to persistent high risks of infection and malignancy. Large cohort studies report cytomegalovirus (CMV) reactivation in 40–60% of kidney recipients without prophylaxis, and even with prophylaxis the rate remains 15–25% (5); BK polyomavirus (BK) viraemia occurs in about 15–20%, and 5–10% of those progress to BK virus nephropathy and graft loss (6). The cumulative incidence of post-transplant lymphoproliferative disorder (PTLD) in adult kidney transplantation is 0.5–2%, but can rise to 5–10% in children and EBV-seronegative recipients (7). Skin cancer is the most frequent malignancy; kidney transplant recipients have a 65- to 100-fold higher risk of squamous cell carcinoma and a >200-fold higher risk of Kaposi’s sarcoma than the general population (8, 9). More notably, the cumulative incidence of non-melanoma skin cancer reaches 40–60% at 20 years post-transplant (9). Metabolically, new-onset diabetes caused by tacrolimus and cyclosporine occurs in 10–20% of patients at 1 year post-transplant, accompanied by accelerated atherosclerosis, and cardiovascular events are the leading cause of death in recipients with normally functioning grafts (10). The burden of lifelong medication also significantly lowers quality-of-life scores (the Short Form-36 (SF-36) mental component summary score is 0.5–1 standard deviation below the general population) and contributes to non-adherence, which is implicated in about 30% of late acute rejections (11).

Transplant immune tolerance is a state in which the recipient maintains normal donor-graft function without maintenance immunosuppressants, while retaining intact immune responses to third-party antigens. Clinically, “operational tolerance” is usually defined as discontinuation of all immunosuppressants for more than 1 year, with no acute rejection, stable graft function, and no subclinical rejection on biopsy (12). Worldwide, the number of reported operational tolerance cases in kidney transplantation is estimated to be fewer than 300–500, most of which were discovered incidentally after unplanned cessation or non-adherence; prospectively induced cases are far rarer (13). Operational tolerance is more common in liver transplantation; large multicentre studies indicate that, under stringent selection, about 40–50% of long-term liver recipients can withdraw all drugs (14). This difference stems from the liver’s inherent immune privilege and regenerative capacity (15–17). Complete tolerance – defined as no chronic graft injury without any immune intervention – has been observed only in some successful mixed-chimerism protocols (18). The long-term clinical value of tolerance induction extends beyond eliminating drug toxicity; it also reduces chronic rejection. Modelling suggests that if 50% of kidney recipients could be converted to tolerance, median graft survival might increase from the current 10–12 years to over 20 years, substantially reducing the need for retransplantation and easing healthcare system burdens.

Sequential therapy for tolerance induction was first systematically articulated by Calne in 1998 with his “Prope tolerance” concept (19, 20), using a single perioperative 30-mg dose of anti-CD52 monoclonal antibody (alemtuzumab) to deplete peripheral lymphocytes profoundly, followed by low-dose tacrolimus (trough 5–8 ng/mL), with the expectation that recipients would enter a state of “almost no need for immunosuppression.” Later, Kirk and colleagues showed in non-human primate kidney transplant models that sequential anti-CD154 plus CTLA4-Ig could induce stable tolerance, with no rejection for more than 5 years after drug cessation (21). This discovery spurred the clinical development of the costimulation blocker belatacept (CTLA4-Ig) (22). In recent years, with the development of Fc-engineered anti-CD40 monoclonal antibodies (23), regulatory cell therapies (24), and non-myeloablative bone marrow transplantation techniques (18, 25), have shaped a complete “sequential antibody induction” paradigm. During the high-risk immune window around transplantation, antibodies precisely block key pathways or delete reactive clones; then immunosuppressants are tapered or switched to guide immune reconstitution toward a regulatory phenotype, eventually allowing partial or complete withdrawal. The core principle is to concentrate active immune interventions early to achieve durable immune resilience, rather than relying on lifelong non-specific suppression.have shaped a complete “sequential antibody induction” paradigm. During the high-risk immune window around transplantation, antibodies precisely block key pathways or delete reactive clones; then immunosuppressants are tapered or switched to guide immune reconstitution toward a regulatory phenotype, eventually allowing partial or complete withdrawal. The core principle is to concentrate active immune interventions early to achieve durable immune resilience, rather than relying on lifelong non-specific suppression.

Despite several sequential antibody protocols entering clinical trials, they show considerable heterogeneity in operational tolerance rates, infection profiles, and cost-effectiveness. For example, complete withdrawal rates are only about 10–20% with T-cell depletion alone, whereas mixed-chimerism protocols can reach 50–70%, but their conditioning toxicity and complexity limit them to very few recipients. In this review, we define “feasibility” across five dimensions: (i) efficacy (operational tolerance rate), (ii) safety (infection, donor-specific antibody (DSA), malignancy), (iii) operational complexity (technical requirements and monitoring), (iv) economic cost (drugs, cell preparation, hospitalisation), and (v) ethical/regulatory aspects (patient selection and informed consent). We aim to dissect the immunological basis, systematically review the evidence from major clinical protocols, and perform a comprehensive feasibility assessment. Finally, we propose directions to overcome translational bottlenecks, providing a reference for moving the transplant community from lifelong immunosuppression to controlled tolerance.

## Mechanistic basis and therapeutic strategy framework for immune tolerance induction

### Significance of sequential treatment strategies

The purpose of sequential therapy is to improve long-term graft survival and to reduce the risk of graft dysfunction and complications that arise when antibody monotherapy fails to induce tolerance. After living-donor kidney transplantation, donor-derived passenger leukocytes migrate in large numbers and activate recipient T cells within 1–2 weeks, creating a peak of immune activation (26). Sequential protocols use high-efficacy antibodies with short half-lives [e.g., alemtuzumab 30 mg single dose depletes lymphocytes for 2–4 weeks; anti-thymocyte globulin (ATG) has a half-life of about 5–14 days (27)] immediately before or at transplantation to cover this window. Subsequently, during the lymphocyte recovery phase, targeted drugs (e.g., belatacept given intravenously every 4 weeks to maintain steady-state concentrations) or low-dose immunosuppressants guide nascent T cells toward a regulatory phenotype. The clinical timeline must address three phases: the antibody storm period (0–3 months) for deep depletion or blockade; the reconstitution window (3–12 months) to shape a low-reactivity repertoire using the drug environment; and the consolidation phase (>12 months) for gradual withdrawal, testing the immune balance. Premature withdrawal may allow donor-reactive memory T cells to reoccupy the immune space, while delayed withdrawal may lead to drug dependence and miss the window for regulatory network maturation. Thus, a successful sequential protocol must, like programming, complete depletion, modulation, and consolidation in the correct order.

### Core mechanisms of transplant immune response and rejection

**Figure 1:**
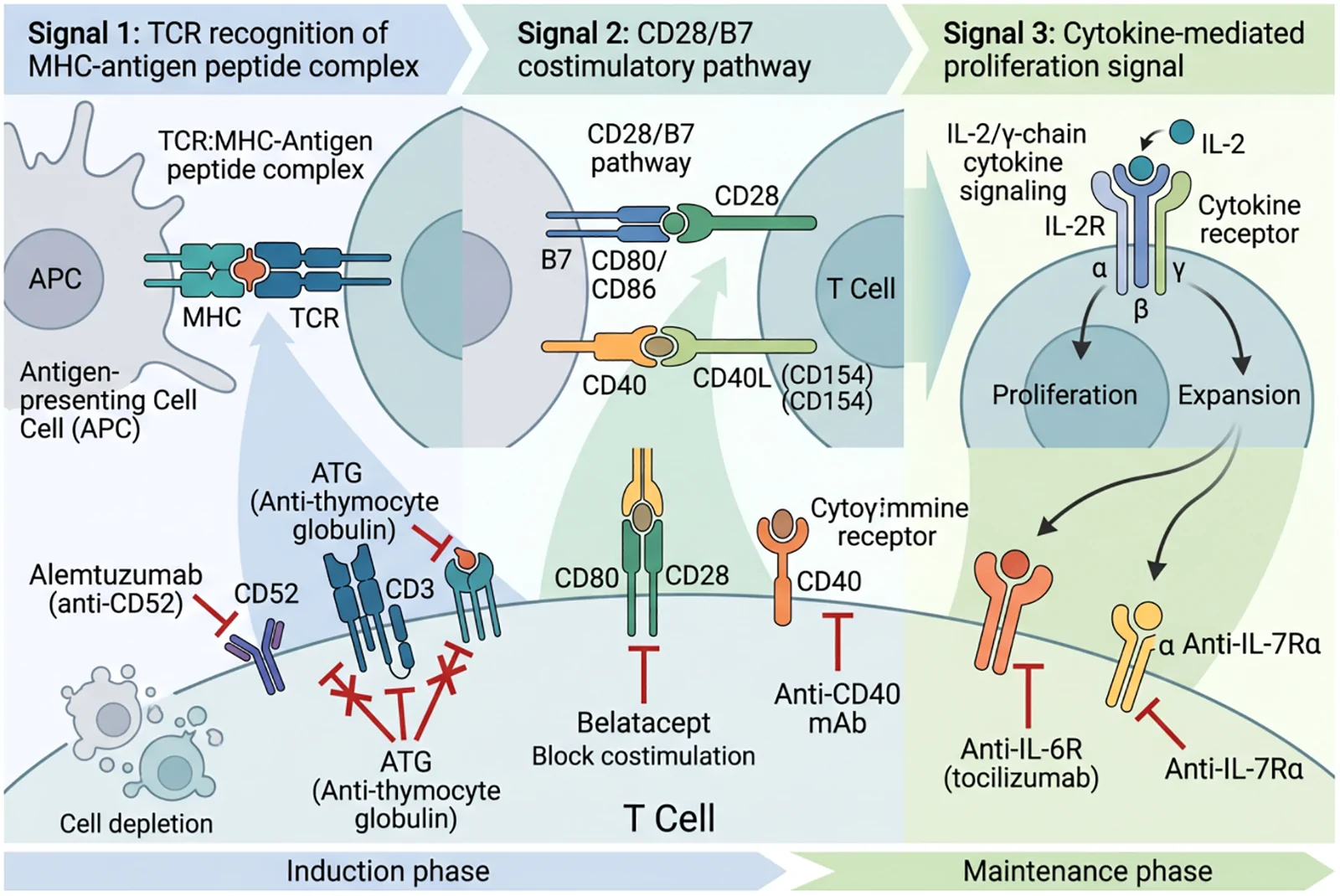
Key targets for sequential antibody intervention based on the three-signal model of T-cell activation. Signal 1: TCR–MHC/peptide recognition, targeted by anti-CD52 (alemtuzumab) or ATG (depletion). Signal 2: CD28–B7 costimulation, targeted by belatacept (CTLA4-Ig) and anti-CD40 (iscalimab). Signal 3: IL-2-driven expansion, targeted by anti-IL-6R (tocilizumab) and anti-IL-7Rα. The figure distinguishes the induction period (deep depletion/blockade) from the maintenance period (regulatory network reconstruction), illustrating the transition from immunosuppression to active tolerance.

Allograft rejection is primarily triggered by recipient T cell recognition of donor major histocompatibility complex (MHC) (28). Full T-cell activation depends on a finely regulated three-signal model. Signal 1 is the specific recognition of donor MHC–peptide complexes by the T-cell receptor (TCR); this signal is antigen-specific but alone insufficient to drive effector functions. Signal 2 is provided by B7 family molecules (CD80/CD86) on antigen-presenting cells (APCs) binding to CD28 on T cells – the key costimulatory signal. Signal 3 is mediated by γ-chain cytokines such as IL-2, which drive clonal expansion and effector differentiation. If Signal 2 is blocked early after transplantation, T cells become anergic, showing hyporesponsiveness to restimulation; this mechanism underpins belatacept-based strategies (29). In addition, costimulation blockade can selectively promote regulatory T-cell (Treg) proliferation (30). After 6 months of continuous belatacept treatment, the proportion of Tregs (CD4^+^ CD25^hi^ CD127^lo^) in peripheral blood increases about 1.5- to 2.0-fold relative to baseline, while effector T-cell proportions remain stable or decrease, thereby increasing the Treg/Teff ratio (31). Sequential antibody regimens precisely modulate these three signals in a spatiotemporal manner (**Figure 1**).

### Basic types of immune tolerance

Central tolerance – clonal deletion of donor-reactive immature T cells in the thymus – is mainly achieved through mixed-chimerism strategies. When donor haematopoietic stem cells engraft, donor-derived dendritic cells migrate to the thymus and mediate negative selection of donor-reactive T cells (32). Studies show that after non-myeloablative conditioning with bone marrow infusion, if donor chimerism remains >1% for more than 6 months, about 80–90% of recipients can successfully stop immunosuppressants, with thymic deletion playing a central role (18). Peripheral tolerance operates through multiple synergistic mechanisms: mature T cells undergo apoptosis or anergy in the absence of costimulatory signals; immune deviation shifts the response from Th1 to Th2, reducing graft inflammation; and active suppression by Tregs is a key execution pathway. In tolerant kidney recipients, the proportion of FOXP3^+^ T cells in peripheral blood is often higher than in stable medicated patients, and the intragraft Treg/CD8 ratio is increased 2- to 3-fold, indicating a local immunoregulatory microenvironment (33).

### Key mechanisms of tolerance induction by antibodies

Depletion and reconstitution is a primary mechanism. Alemtuzumab (anti-CD52) and ATG target CD52 and various T-cell surface molecules, reducing peripheral lymphocyte counts by >95% within hours. CD4^+^ T-cell depletion is more profound, and recovery to 50% of pre-transplant levels typically takes 12–24 months (27). In severe lymphopenia, T-cell reconstitution driven by thymic output and homeostatic proliferation exhibits biased hyporesponsiveness to donor antigens; if continuous donor antigen exposure occurs during this period, donor-specific deletion or anergy is readily induced (34). Immunological analysis of kidney recipients who discontinued medication after alemtuzumab induction showed that the frequency of donor-specific IFN-γ-secreting T-cell clones decreased by about 70–90%, with particularly marked hyporesponsiveness in the indirect recognition pathway (35). Costimulation blockade is another critical pathway. Belatacept has about 2–4 times higher affinity for B7 molecules than CTLA-4 (KD ≈ 20 nM vs. 50–100 nM), effectively reversing CD28-B7 signalling (22). Anti-CD40 monoclonal antibodies block the CD40-CD154 pathway, which not only supports T-cell help for B-cell antibody production but also sustains germinal centre reactions (23). After 12 weeks of iscalimab (anti-CD40) treatment, plasmablast and memory B-cell proportions in the blood of kidney recipients decrease by about 30% and 20%, respectively, and the pro-inflammatory cytokine IL-21 level falls by nearly 50%, significantly inhibiting *de novo* DSA production (36). Importantly, anti-CD40 antibodies suppress follicular helper T cells (Tfh), which are critical for germinal centre formation and DSA generation; by inhibiting Tfh, these antibodies reduce DSA (36). Regulatory network reinforcement also plays a key role. Low-dose anti-CD3 monoclonal antibody (teplizumab) preferentially expands IL-10-secreting regulatory cells and increases FoxP3 expression, a Treg marker (37). In preclinical models, anti-CD45RB monoclonal antibody increases CD4^+^ CD25^+^ Treg numbers while reducing CD8^+^ memory T-cell function; subsequent donor bone marrow infusion after this induction achieves stable mixed chimerism (38). However, Tregs are unstable in inflammatory environments and can convert to Th17 or effector phenotypes. Strategies to maintain Treg stability include mTOR inhibitors (sirolimus), which promote Treg survival and function, and low-dose IL-2 to sustain FoxP3 expression (39). Finally, immune deviation is induced when apoptotic cell clearance promotes regulatory macrophages and immature dendritic cells (DCs) that secrete IL-10 and transforming growth factor-β (TGF-β), creating a tolerogenic milieu. This “regulatory clearance” process itself has tolerance-inducing properties and does not require additional immunosuppression (40).

### Transition of immunosuppressants from “immune coverage” to “active modulation”

In sequential protocols, the role of immunosuppressants shifts from non-specific “immune coverage” to active assistance in tolerance induction. Mycophenolate mofetil (MMF), by inhibiting inosine monophosphate dehydrogenase, selectively blocks the *de novo* purine synthesis pathway in lymphocytes. Its intensity is sufficient to suppress activated T and B cells but has only a minor effect on Treg survival, as Tregs rely more on the salvage pathway (41). Immunophenotyping from clinical trials confirms that Treg proportions can be maintained or even increased under MMF maintenance (42). The mTOR inhibitor sirolimus, by blocking post-IL-2R signalling, selectively promotes thymic output and peripheral expansion of Tregs (39). Switching to a low-dose sirolimus-containing regimen increases the peripheral Treg proportion from 4.1% to 7.8% while inhibiting Th17 differentiation, creating a more favourable regulatory/effector balance (43). Low-dose tacrolimus (trough 3–5 ng/mL), compared with full-dose (8–12 ng/mL), has weaker inhibition of TCR-downstream calcineurin signalling, preserving some activation-induced apoptotic signals and thus helping to delete donor-reactive clones rather than merely suppressing them (44). This fine-tuned synergy underlies the higher success rates of sequential protocols over antibody induction alone.

### Major clinical sequential antibody tolerance induction protocols and evidence

T-cell depletion regimens with alemtuzumab or ATG allow about 11–20% of kidney recipients to achieve complete drug withdrawal, but they carry risks of subclinical AMR, DSA generation, and infections (e.g., CMV, PTLD) (45). Costimulation-blockade protocols using belatacept with basiliximab or anti-CD40 (iscalimab) can significantly improve renal function, reduce DSA incidence, and enable CNI-free or dual-agent maintenance in some low-risk recipients (46). Non-myeloablative bone marrow transplantation combined with thymic or total lymphoid irradiation induces stable mixed chimerism in 50–80% of recipients, allowing long-term drug-free tolerance, but the conditioning is complex and high-risk (18, 25). Adoptive transfer of regulatory T cells or regulatory dendritic cells, combined with antibody induction, can safely reduce immunosuppression intensity and lower acute rejection and infection rates, but complete withdrawal remains uncommon (24). Novel antibodies – anti-CD45RB, anti-LFA-1, anti-IL-6R, and anti-IL-7Rα – show promise in preclinical or early-phase studies (38, 47, 48). Overall, the protocols differ markedly in efficacy, safety, and operational complexity, with complete tolerance rates ranging from about 10% to 70%, and no single regimen is universally applicable. **Table 1** summarises the major protocols, with added columns for sample size, follow-up, and organ type.

**Table 1:** Major clinical sequential antibody tolerance induction protocols and evidence.

Protocol category	Specific protocol/representative study	Key drugs/interventions	Sample size (n)	Follow-up	Organ type	Efficacy (tolerance/withdrawal rate)	Safety/main issues
T-Cell Depletion	Calne team alemtuzumab induction (1998)	Alemtuzumab 30 mg ×2 doses + low-dose tacrolimus monotherapy	**117**	**5 yrs**	**Kidney**	Complete withdrawal >1 year in ~12.8% (15/117); ~20% converted to every-other-day or twice-weekly dosing	Subclinical AMR signs in 30%, DSA positivity at 3 years 18% (20, 49-50)
T-Cell Depletion	Nocera et al. ATG sequential protocol	Rabbit anti-thymocyte globulin (rATG) (2 mg/kg ×5d) + MMF + tacrolimus, reduced to very low dose at 3 months	**45**	**2 yrs**	**Kidney**	Complete withdrawal (MMF only) in 17.8% (8/45); 84% maintained low-dose	CMV viraemia 41%, PTLD 8.9%; slow CD4 recovery (51)
Costimulation Blockade	Berlin centre belatacept + basiliximab induction, rapid CNI withdrawal	Belatacept + basiliximab, stop CNI at 3 months, maintain belatacept+MMF	**20 (single-arm)**	**1 yr**	**Kidney**	65% maintained CNI-free, 20% converted to belatacept monotherapy; 1-year interstitial fibrosis score significantly lower than CNI group	Some resumed CNI due to acute rejection (52)
Costimulation Blockade	Iscalimab (anti-CD40) combined with belatacept and short-term tacrolimus	Iscalimab + belatacept + tacrolimus (stopped at 3 months)	**12**	**6 mo**	**Kidney**	At 6 months, 66.7% (8/12) achieved dual-blockade monotherapy without rejection	No thrombotic events; DSA significantly lower than tacrolimus group (36)
Mixed Chimerism	Massachusetts General Hospital (MGH) protocol	Cyclophosphamide + anti-CD2 + thymic irradiation + donor bone marrow	**10**	**>15 yrs**	**Kidney**	Mixed chimerism in 70% (7/10), all withdrawn; longest follow-up >15 years	Conditioning complex, requires thymic irradiation or anti-CD154 (latter withdrawn due to thrombosis) (18, 53); **late acute rejection ~5-10% after chimerism loss** (54)
Mixed Chimerism	Stanford University protocol	Total lymphoid irradiation + ATG + donor CD34^+^ cells/Treg infusion	**24**	**36 mo**	**Kidney**	66.7% (16/24) completely withdrawn, normal renal function	Depends on high CD34^+^ and Treg proportion; **late acute rejection ~5-10% after chimerism loss** (25, 54)
Cell Therapy	ONE Study (multicentre Treg adoptive transfer)	Polyclonal Treg + anti-CD25 induction	**38**	**1 yr**	**Kidney**	1-year acute rejection 10.5% vs. control 12.5% (NS); 55% of recipients had tacrolimus trough 2-4 ng/mL	Complete withdrawal rare; need to improve cell persistence (24)
Cell Therapy	ARTEMIS trial (darTreg)	Donor-antigen reactive Treg	**–**	**6 mo**	**Kidney**	59.8% achieved very low CNI at 6 months; 52.4% low-dose mono/dual maintenance	~2.4-fold improvement over historical controls; larger trials ongoing (55)
Cell Therapy	DCreg Phase 1 trial	Donor-derived regulatory DCs + basiliximab + tacrolimus/MMF	**16**	**2 yrs**	**Kidney**	2-year rejection 0%; donor-specific IFN-γ^+^ T cells decreased by 40%, IL-10^+^ T cells increased	Small sample (n=16), high production cost, complex quality control (56)
Other Novel Targets	Anti-CD45RB + CTLA4-Ig (non-human primate)	Anti-CD45RB mAb + CTLA4-Ig	**6 (monkeys)**	**>500 days**	**NHP kidney**	All 6 rhesus macaques achieved >500 days drug-free tolerance	Humanised version not in Phase III (38)
Other Novel Targets	Tocilizumab (anti-IL-6R) as adjunct for chronic AMR	Tocilizumab + belatacept/low-dose tacrolimus	**–**	**–**	**Kidney**	DSA mean fluorescence intensity decreased 30-40%, estimated glomerular filtration rate (eGFR) stable	Prospective trial (NCT04536298) ongoing (48, 57)

Data in this table were verified against the corresponding source publications and main text. All percentages and sample sizes are consistent with the reported studies. For the ARTEMIS trial (darTreg), the sample size was not separately reported in the available sources; the efficacy data are derived from the published trial results (55). For the tocilizumab adjunctive therapy, DSA mean fluorescence intensity reduction and eGFR stabilization data are from Choi et al. (48), with the prospective trial NCT04536298 ongoing (57). All studies listed are in kidney transplantation unless otherwise indicated; NHP, non-human primate. Bold values denote the sample size, follow‑up duration, and organ type for each study.

## Alemtuzumab induction followed by low-dose monotherapy maintenance and withdrawal trials

Calne’s team first reported in 1998 that 31 deceased-donor kidney recipients received alemtuzumab 30 mg on the day before and 24 hours after transplantation (two doses), followed by low-dose tacrolimus monotherapy (trough 5–8 ng/mL) (20). At a mean follow-up of 24 months, 29 had functioning grafts, 10 (32%) achieved “micro-dose maintenance” with tacrolimus given every 48 hours or even less frequently, but only 2 withdrew completely. In an extended cohort of 117 recipients, 5-year graft survival was 85%, and about 20% could be converted to every-other-day or twice-weekly dosing, but only 15 (12.8%) achieved complete withdrawal for >1 year (49). Notably, protocol biopsies revealed that about 30% of low-dose maintenance recipients had subclinical AMR signs (microvascular inflammation or C4d deposition), and DSA positivity rose from 0% pre-transplant to 18% at 3 years (58). Thus, T-cell depletion alone, while substantially reducing the need for immunosuppression, inadequately controls B-cell and antibody responses, and the long-term risk of chronic graft injury cannot be overlooked (50, 59).

## ATG sequential mycophenolate/tacrolimus reduction protocol

**Figure 2:**
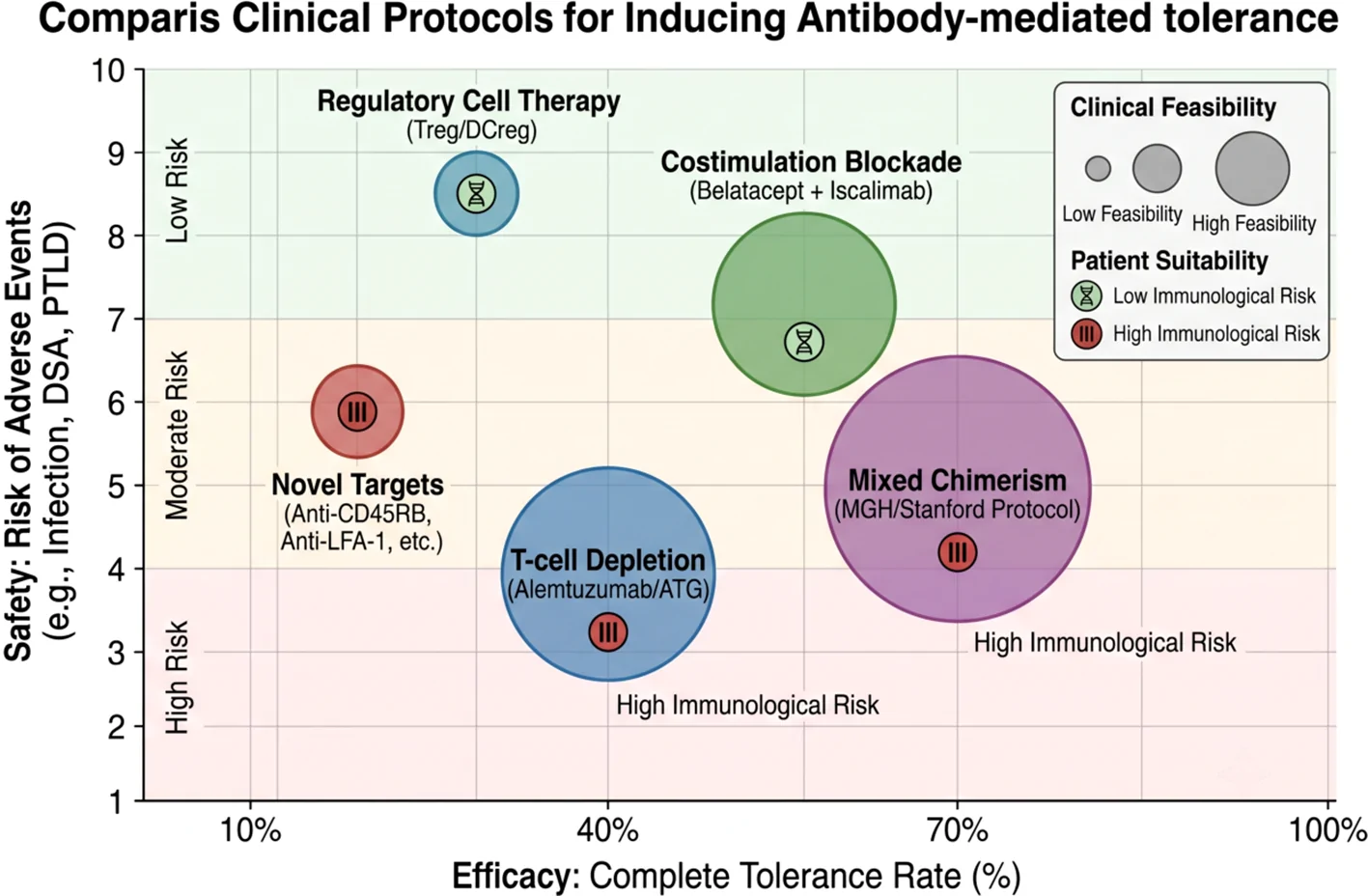
Comparisons of clinical efficacy and feasibility of major sequential antibody tolerance induction protocols. This figure is a multidimensional comparison diagram that aims to systematically compare the clinical efficacy and feasibility of the main sequential antibody tolerance induction schemes. The included schemes are: 1) T cell depletion (alemtuzumab/ATG), 2) co-stimulation blockade (belatacept with or without iscalimab), 3) mixed-chimeric body (MGH and Stanford), 4) regulatory cell therapy (Treg/DCreg adoptive transfer), and 5) novel targets (e.g., anti-CD45RB, anti-LFA-1). The comparison dimensions include complete tolerance rate (from 10% to 70%), safety (infection, DSA generation, and PTLD incidence), operational complexity (whether bone marrow pretreatment, cell preparation, or special equipment is required), and applicability (low- or high-immunological-risk recipients). The figure uses radar or bubble diagrams to illustrate efficacy, with the x-axis representing safety and the y-axis representing operational complexity. The size of the bubbles or the length of the radar axes represents applicability. This figure intuitively reveals the advantages, limitations, and balance points of different schemes in the process of converting to “clinically applicable tolerance induction”.

A representative approach using rATG (2 mg/kg for the first 5 days post-transplant) combined with MPA and tacrolimus, with gradual reduction of tacrolimus to very low trough levels (2–4 ng/mL) after 3 months, has been explored in living-donor kidney transplantation. In a cohort of 45 recipients, 38 (84%) successfully maintained this low-dose regimen, and 8 (17.8%) completely discontinued tacrolimus at 2 years post-transplant, maintained on MPA alone without rejection for >1 year (51). However, CMV DNAemia occurred in 41%, and 4 recipients (8.9%) developed PTLD, highlighting the sustained immune surveillance defect from profound lymphocyte depletion. T cell subset analysis showed that the median time for CD4^+^ T cells to recover to >200/μL was 18 months, while CD8^+^ T cells recovered faster, leading to long-term CD4/CD8 inversion, likely a major factor in the increased PTLD risk. The 1-year acute rejection rate for lymphocyte depletion protocols is 15–25%, mostly occurring during dose reduction or after infection triggers. A meta-analysis of 260 kidney transplant recipients receiving alemtuzumab induction showed a pooled proportion of complete tolerance (withdrawal of all immunosuppressants >1 year) of 11% (95% CI 7–16%) at 1 year post-transplant (45). Heterogeneity was significant (I²=72%), reflecting differences in patient selection and maintenance protocols (**Figure 2**). Among acute rejection rebounds, 80% were steroid-sensitive, but 5–10% still required ATG or resumption of full-dose CNI. The long-term risk of graft function decline after rebound rejection is not negligible; recipients who experienced rebound had a 5-year eGFR about 8–10 mL/min/1.73m² lower than those without rebound.

## Belatacept sequential conversion to CNI reduction strategy

Two Phase 3 trials, BENEFIT and BENEFIT-EXT, enrolled a total of 1209 kidney recipients and confirmed that the belatacept group had significantly higher eGFR than the cyclosporine group at 1, 3, and 7 years (mean eGFR difference at 7 years about 10–12 mL/min/1.73m^2^), and DSA incidence was only one-third that of the cyclosporine group (5% vs. 15%) (46, 60, 61). The 5-year results of BENEFIT were reported by Rostaing et al. (62), and the 7-year final outcomes of BENEFIT-EXT by Durrbach et al. (60). Based on this evidence, several centres have tested tolerance-directed protocols using belatacept plus basiliximab (anti-CD25) induction with rapid CNI withdrawal. In a representative single-arm trial of 20 low-risk living-donor kidney recipients, basiliximab and belatacept were given at transplantation, and CNI was stopped at 3 months, with maintenance on belatacept and mycophenolate. At 1 year, 13 (65%) remained CNI-free, and 4 (20%) subsequently reduced or stopped mycophenolate to achieve belatacept monotherapy (52). Biopsies showed that those who successfully discontinued CNI and remained on belatacept monotherapy had significantly lower 1-year interstitial fibrosis scores (ci score) than controls continuing CNI (0.3 ± 0.4 vs. 1.1 ± 0.7, P = 0.008). Note: this is a single-arm, small-sample trial (n=20), so its evidence level is exploratory. These data suggest that belatacept-based sequential protocols have the potential to induce markedly reduced or even monotherapy tolerance in some recipients, with clear histological advantages.

## Early clinical exploration and challenges of anti-CD40/CD154 monoclonal antibody sequential therapy

Anti-CD154 monoclonal antibody (ruplizumab) showed powerful tolerance-inducing ability in early non-human primate studies; combined with donor bone marrow and short-term CNI, 10 of 12 cynomolgus monkeys achieved >500 days drug-free survival (21). However, a Phase 2 clinical trial was halted in 2001 due to three thromboembolic events (1 myocardial infarction, 2 pulmonary embolisms), later confirmed to be caused by Fc-mediated platelet activation via FcγRIIa binding (53). Fc-engineered, silent (Fc-silent) anti-CD40 monoclonal antibodies were subsequently developed (23). In a Phase 2 trial of iscalimab (CFZ533), 85 kidney transplant recipients were randomized to iscalimab- or tacrolimus-based regimens, both with MPA. Results showed that the 6-month clinical rejection rate in the iscalimab group was comparable to the tacrolimus group (10.3% vs. 7.5%), but estimated GFR was significantly higher (77.6 vs. 65.3 mL/min/1.73m², P = 0.02), DSA incidence was significantly lower (2.6% vs. 12.5%), and no thrombotic events were observed (36). Based on this, a tolerance-directed trial further used iscalimab combined with belatacept and short-term tacrolimus (discontinued at 3 months post-transplant). Preliminary data from 12 recipients showed that at 6 months, 8 (66.7%) achieved maintenance on belatacept and iscalimab dual-blockade alone without rejection (36). This direction may be one of the most promising pathways to achieve CNI-free tolerance in the near future. In mouse and non-human primate experiments, simultaneous blockade of the CD28/B7 and CD40/CD154 pathways induced deep and durable donor-specific tolerance, with the Treg/Teff ratio in tolerant grafts increasing 5- to 10-fold (30). Mechanistically, dual blockade not only synergistically promotes T cell anergy but also strongly inhibits germinal center reactions and plasma cell generation, fundamentally preventing DSA production. A Phase 2/3 trial is currently evaluating the feasibility and long-term effects of belatacept combined with anti-CD40 monoclonal antibody (iscalimab) for sequential withdrawal of tacrolimus in kidney transplantation, with the expectation that some recipients will achieve dual-blockade monotherapy maintenance within 18–24 months, which would greatly broaden the applicable population for tolerance induction.

## Non-myeloablative bone marrow transplantation combined with T cell-depleting antibodies and thymic irradiation

The Massachusetts General Hospital (MGH) protocol used conditioning with cyclophosphamide, anti-CD2 monoclonal antibody, and thymic irradiation, followed by donor bone marrow infusion. Among 10 HLA-mismatched kidney transplant recipients, 7 (70%) established mixed chimerism (donor cells 1–30%), and all 7 successfully discontinued all immunosuppressants, with the longest follow-up exceeding 15 years without rejection (18). A subsequent modified protocol removed thymic irradiation and replaced it with anti-CD154 monoclonal antibody; results showed that 4 of 5 (80%) established chimerism and withdrew, with 2 of them, after discontinuation of anti-CD154, switching to belatacept maintenance, and also followed >5 years without rejection (63). These cases provide the strongest evidence for clinical complete tolerance. However, ~5-10% of tolerant recipients may experience late acute rejection after chimerism loss, requiring prolonged monitoring (54). The Stanford protocol used total lymphoid irradiation (TLI) plus ATG conditioning, followed by infusion of donor haematopoietic stem cells containing CD34^+^ cells and Tregs, with post-transplant tacrolimus and MMF maintenance. Among 24 living-donor kidney recipients, 16 (66.7%) had completely stopped immunosuppressants at 36 months, with normal renal function (25). No severe infections or malignancies occurred after withdrawal. Success was closely associated with higher CD34^+^cell numbers (median 8.2×10^6^/kg) and Treg proportions (CD4^+^ CD25^hi^ gh >50%) in the infused product. Achieving donor chimerism >1% for at least 6 months is a reliable predictor of complete drug withdrawal, with sensitivity >85% and specificity 100% (18). Long-term follow-up shows that once chimerism is completely lost, latent alloreactive memory T cells may reactivate, leading to late acute rejection in about 5–10% of tolerant recipients, necessitating re-initiation of immunosuppression (54). Thus, chimerism strategies require extended monitoring.

## Adoptive transfer of regulatory T cells combined with anti-CD25 or low-dose cyclophosphamide induction

The ONE Study – a multicentre European pilot involving the UK, Germany, and Italy – tested polyclonal Treg adoptive transfer with anti-CD25 induction. Pooled data from 38 Treg-treated patients showed a 1-year acute rejection rate of 10.5%, compared with 12.5% in the standard control group (not significant). However, the Treg group had significantly lower immunosuppressant doses: 55% achieved tacrolimus troughs of 2–4 ng/mL, versus only 15% in controls (P<0.01) (24). A similar approach using donor-antigen reactive Tregs (darTregs) was investigated in a ONE Study consortium pilot trial, demonstrating feasibility and safety, and supporting CNI minimisation (55). Pooled data from 48 patients across ONE Study arms [Treg, DCreg, regulatory macrophages (Mreg)] showed no significant difference in 1-year eGFR compared with standard immunosuppression (71 vs. 68 mL/min), but slightly lower biopsy-proven acute rejection (8.3% vs. 12.5%) and fewer serious infections (7 vs. 15 cases) (24). Cell therapy with antibody induction is safe and helps reduce overall immunosuppression intensity. However, complete withdrawal remains rare, and next steps must improve cell persistence and specificity.

## Combination of donor-derived regulatory dendritic cells and antibody costimulation blockade

In a first-in-human Phase 1 trial, 16 living-donor kidney recipients received a single pre-transplant infusion of donor-derived regulatory dendritic cells (DCregs, generated *in vitro* with vitamin D3 and IL-10), combined with basiliximab and tacrolimus/MMF maintenance (56). No serious infusion-related adverse events occurred, and the 2-year rejection rate was 0%. Circulating donor-specific IFN-γ^+^ T-cell frequency decreased by about 40%, while IL-10^+^ T cells increased. Single-cell RNA sequencing revealed that monocytes in the peripheral blood of DCreg recipients shifted toward an anti-inflammatory phenotype, and Treg expansion occurred. This strategy is now being studied sequentially with belatacept to enhance drug-withdrawal tolerance (56). Pooled data from 48 patients across ONE Study arms (Treg, DCreg, Mreg) showed no significant difference in 1-year eGFR compared with standard immunosuppression (71 vs. 68 mL/min), but slightly lower biopsy-proven acute rejection (8.3% vs. 12.5%) and fewer serious infections (7 vs. 15 cases) (24). Cell therapy with antibody induction is safe and helps reduce overall immunosuppression intensity. However, complete withdrawal remains rare, and next steps must improve cell persistence and specificity.

## Combinations of adhesion molecule antibodies such as anti-CD45RB and anti-LFA-1

Anti-CD45RB monoclonal antibody (MB20-11) in a rhesus macaque kidney transplant model, when combined with CTLA4-Ig, enabled all 6 recipients to achieve >500 days of drug-free tolerance with normal graft histology (38). Mechanistic studies showed that anti-CD45RB selectively reduces CD8^+^ central memory T cells without affecting Tregs. However, a humanised version has not entered clinical use. Anti-LFA-1 (efalizumab) was withdrawn in 2009 because of an increased risk of progressive multifocal leukoencephalopathy (PML) in transplant patients, but a new generation of humanised, low-affinity Fc-modified products is in early clinical evaluation, aiming to retain efficacy while reducing safety risks (47).

## Adjunctive value of cytokine-targeted antibodies in suppressing memory T cells and AMR

**Figure 3:**
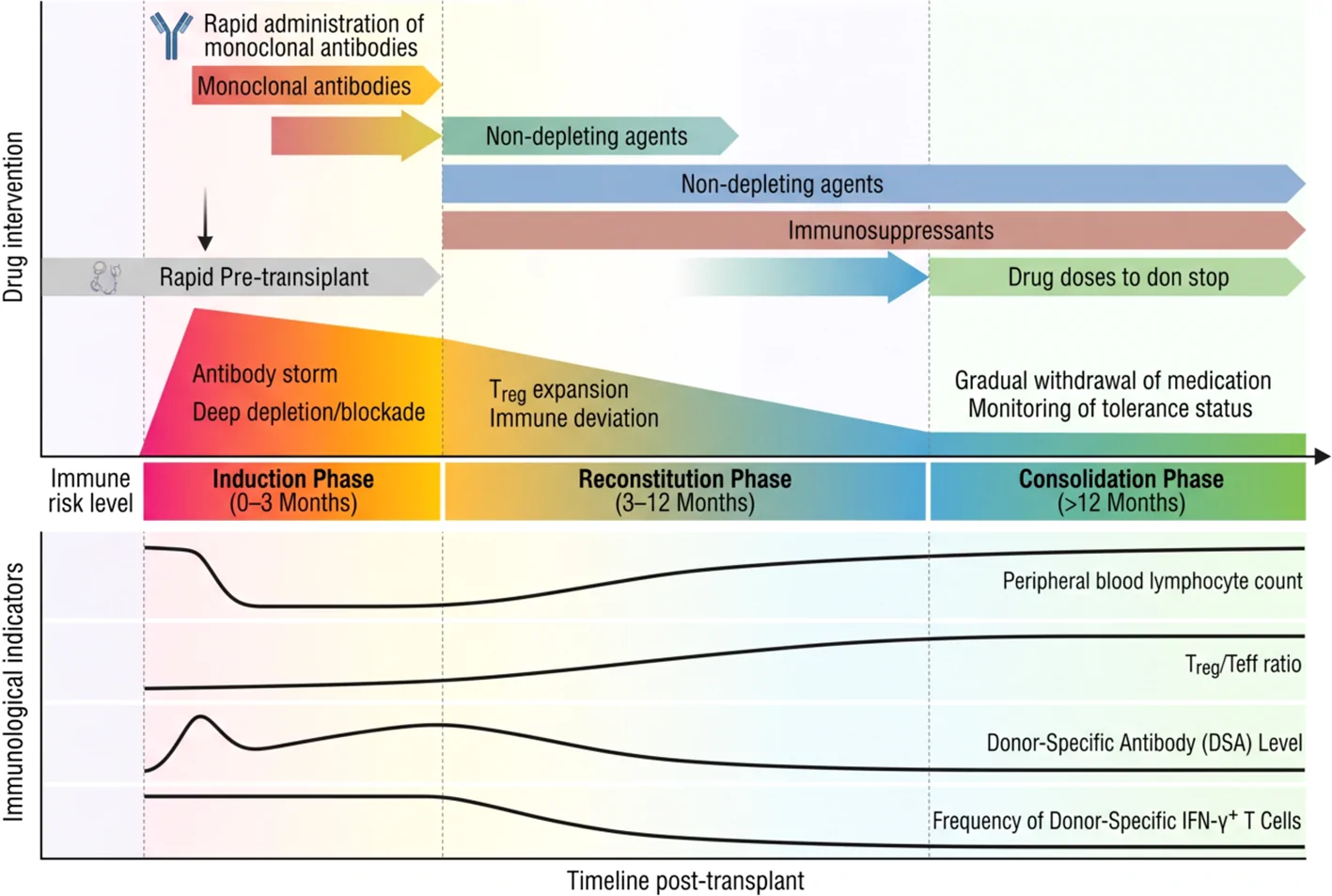
This figure is a chronological schematic diagram that systematically illustrates the dynamic process of immune intervention and immune reconstitution during sequential antibody-mediated tolerance induction therapy, from the pre-transplant period through 12 months post-transplant. The treatment process is divided into three key phases: the induction phase (0–3 months post-transplant), characterized by deep lymphocyte depletion or co-stimulation blockade achieved through an “antibody storm”; the Reconstruction Phase (3–12 months post-transplant), during which newly generated T cells are guided toward a regulatory phenotype under pharmacological conditions, promoting an increase in the Treg/Teff ratio and immune shift; and the Consolidation Phase (12 months post-transplant and beyond), during which immunosuppressants are gradually tapered or discontinued to assess the state of immune balance. The figure overlays multiple immunological indicator curves on a time-axis, including peripheral blood lymphocyte counts (rapid post-operative decline followed by slow recovery), the Treg/Teff ratio (gradual increase), the frequency of donor-specific IFN-γ^+^ T cells (decrease), and DSA levels (requiring monitoring for potential rebound). The combination of a stratified timeline and trend lines for these indicators visually illustrates the spatiotemporal logic of the sequential strategy’s evolution from “immune clearance” to “immune regulation” and then to “tolerance maintenance,” providing a visual basis for determining the timing of drug withdrawal and monitoring milestones in clinical practice.

Tocilizumab (anti-IL-6R) has been used to treat chronic AMR; data show that after 6 months, DSA mean fluorescence intensity decreases by about 30–40% and eGFR stabilises (48). Its sequential use in belatacept/low-dose tacrolimus protocols aims to prevent *de novo* DSA. A prospective trial (NCT04536298) is currently recruiting 120 high-immunological-risk recipients to evaluate its adjunctive value for tolerance induction (57). In addition, anti-IL-7Rα monoclonal antibody (GSK2618960), by blocking IL-7 signalling, reduces homeostatic proliferation of memory T cells. A Phase 1 trial confirmed that it selectively reduces CD4^+^ and CD8^+^ memory T-cell numbers by about 30% *in vivo* without affecting Tregs (64), making it a promising adjunct to depletion protocols (**Figure 3**).

## Design and difficulties of four Novel sequential treatment protocols

The following four protocols are exploratory, based on preclinical or early-phase data; their clinical feasibility remains unproven. **Table 2** summarises their evidence levels. Each protocol description in the main text begins with: “This is an exploratory protocol based on preliminary animal/Phase I data, and its clinical feasibility remains unproven”.

**Table 2:** Summary of four novel sequential tolerance induction protocols with evidence levels.

Protocol	Core drugs/mechanisms	Main advantages	Main problems/risks	Current evidence level
Anti-CD45RB + CTLA4-Ig + Sirolimus (Treg expansion)	Anti-CD45RB mAb, belatacept, sirolimus; selectively depletes CD8^+^ central memory T cells, expands Tregs	Avoids myeloablation; drug-free survival >500 days in NHP	Anti-CD45RB not in Phase III; sirolimus delays wound healing, hyperlipidaemia; human tolerance rate unknown	**Preclinical only** (38, 39)
Anti-IL-7Rα + low-dose anti-CD3 + MMF (memory T-cell modulation)	Anti-IL-7Rα, teplizumab, MMF; reduces memory T cells ~30%, induces regulatory network	Selectively reduces memory T cells without affecting Tregs; suitable for high immunological memory risk	Anti-IL-7Rα in early clinical stage; teplizumab may cause cytokine release; estimated complete withdrawal ~15-25%	**Phase I completed** (37, 64)
Donor DCreg + tocilizumab + short-term low-dose tacrolimus (immune deviation)	Donor DCregs, tocilizumab, tacrolimus; shapes tolerogenic microenvironment, inhibits germinal centre reactions	Avoids deep T-cell depletion; Phase I showed 0% rejection at 2 years, low DSA	DCreg production costly and complex quality control (QC); tocilizumab increases infections and hepatotoxicity; ~30% develop subclinical inflammation after tacrolimus withdrawal	**Phase I completed** (48, 56)
Protocol	Core Drugs/Mechanisms	Main Advantages	Main Problems/Risks	**Preclinical only** (36, 47)

Bold values indicate the level of evidence for each protocol.

## Anti-CD45RB monoclonal antibody combined with CTLA4-Ig and short-term sirolimus for treg expansion sequential protocol

This is an exploratory protocol based on preliminary animal data, and its clinical feasibility remains unproven. The protocol builds on non-human primate evidence that anti-CD45RB (MB20-11) combined with CTLA4-Ig induces long-term drug-free tolerance (38), and also uses sirolimus to selectively expand Tregs (39). The dosing schedule is as follows: Induction period (day –1 to day +7): humanised anti-CD45RB 10 mg/kg i.v. on day –1 (pre-transplant) and days +1, +3, +5, +7 (5 doses total); belatacept (CTLA4-Ig) 10 mg/kg i.v. on day –1 and days +3, +7, +14, +28. Maintenance period (months 1–6): oral sirolimus (loading 6 mg, then 2 mg daily, adjusted to trough 4–8 ng/mL) and belatacept every 4 weeks (10 mg/kg, 5 doses). Withdrawal period (months 6–12): belatacept stopped at month 6; sirolimus tapered by 0.5 mg/day every 4 weeks from months 6–9, then stopped; drug-free observation until month 12. Advantages: selective depletion of CD8^+^ central memory T cells without affecting Tregs; combined costimulation blockade and mTOR inhibition synergistically expand Tregs; avoids myeloablation or bone marrow transplantation; achieved >500 days drug-free survival in animal models (38). Issues: humanised anti-CD45RB has not entered Phase III; safety and immunogenicity are unknown; sirolimus can delay wound healing, cause oral ulcers, and hyperlipidaemia; human tolerance rate not validated; after withdrawal, DSA and Treg/Teff ratio require close monitoring, with urgent resumption of immunosuppression if DSA rises. Current evidence level: Preclinical only (38, 39).

## Anti-IL-7Rα monoclonal antibody combined with low-dose anti-CD3 monoclonal antibody and mycophenolate for memory T cell modulation sequential protocol

This is an exploratory protocol based on preliminary Phase I data, and its clinical feasibility remains unproven. It targets memory T cells and is intended for recipients with high immune memory risk (e.g., retransplantation or young recipients). Anti-IL-7Rα (GSK2618960) blocks IL-7 signalling to reduce memory T-cell homeostatic proliferation (64), while low-dose anti-CD3 (teplizumab) preferentially expands IL-10-secreting regulatory cells (37). Dosing: Induction (from 2 weeks before to 1 week after transplant): anti-IL-7Rα 2 mg/kg i.v. on days –14, –7, 0, and +7 (4 doses); teplizumab 25 μg/kg/day i.v. for 5 consecutive days (days –5 to –1). Maintenance (months 1–9): MMF 750 mg b.i.d. (target trough 2–3 μg/mL) and monthly anti-IL-7Rα on days +28, +56, +84 (3 doses). Withdrawal (months 9–15): anti-IL-7Rα stopped at month 9; MMF tapered by 250 mg/day every 6 weeks from months 9–12, then stopped; drug-free observation for 3 months. Advantages: selectively reduces CD4+ and CD8+ memory T cells by ~30% without affecting Tregs; low-dose anti-CD3 induces a regulatory network; theoretically reduces chronic rejection and DSA; suitable for CNI-refractory high-risk recipients. Problems: anti-IL-7Rα is still early-stage, long-term safety unknown; teplizumab may cause cytokine release syndrome (fever, headache), requiring inpatient monitoring; estimated complete withdrawal only 15–25%; low-dose MMF still carries teratogenicity and infection risks; large-scale human data lacking. Current evidence level: Phase I completed (37, 64).

## Donor-derived regulatory dendritic cells combined with tocilizumab and short-term low-dose tacrolimus for immune deviation sequential protocol

This is an exploratory protocol based on preliminary Phase I data, and its clinical feasibility remains unproven. It is based on the safety and immunomodulatory effects of donor-derived DCregs from the ONE Study and subsequent trials (24, 56), combined with tocilizumab (anti-IL-6R) to inhibit germinal centre reactions and plasma-cell differentiation (48). Schedule: Cell preparation (6 weeks before transplant): CD14^+^ monocytes isolated from the donor and cultured with GM-CSF, IL-4, vitamin D3, and IL-10 for 7 days to generate DCregs. Induction: single i.v. DCreg infusion (2–5×10^6^/kg) on day –7; tocilizumab 8 mg/kg i.v. on day –1 and days +14, +28; oral tacrolimus (trough 3–5 ng/mL) from day 0. Maintenance (months 1–6): continue low-dose tacrolimus and monthly tocilizumab on days +56, +84, +112. Withdrawal: tocilizumab stopped at month 6; tacrolimus target trough reduced by 1 ng/mL every 6 weeks from months 6–9, then stopped; drug-free observation until month 12, with monitoring of peripheral blood IL-10/TGF-β and donor DNA chimerism. Advantages: uses natural regulatory cells to actively shape a tolerogenic microenvironment, avoids deep T-cell depletion, fewer infections; Phase I showed 0% rejection at 2 years and low DSA (56). Problems: DCreg preparation is patient-specific, costly, and quality-control-intensive, difficult to standardise; tocilizumab may increase upper respiratory infections and liver enzyme elevations; after tacrolimus withdrawal, ~30% may develop subclinical inflammation, requiring frequent biopsies; small sample (n=16), long-term durability unclear. Current evidence level: Phase I completed (48, 56).

## Low-affinity Fc-engineered anti-LFA-1 antibody combined with anti-CD40 monoclonal antibody and short-term cyclophosphamide for central memory T cell depletion sequential protocol

This is an exploratory protocol based on preliminary animal data, and its clinical feasibility remains unproven. It uses a new generation of low-affinity, Fc-silent anti-LFA-1 (odulimab) to retain blockade of lymphocyte migration while reducing PML risk (47), combined with anti-CD40 (iscalimab) and low-dose cyclophosphamide to deplete central memory T cells. Dosing: Induction (day –3 to day +14): anti-LFA-1 loading 10 mg/kg i.v., then 5 mg/kg weekly on days –3, 0, + 7, +14 (4 doses); iscalimab 10 mg/kg i.v. on days –1, +7, +14, +28 (4 doses); cyclophosphamide 6 mg/kg i.v. on days –2 and 0 (2 doses). Maintenance (months 1–6): monthly iscalimab 10 mg/kg (5 doses until month 6); no further anti-LFA-1. Withdrawal: all drugs stopped at month 6; drug-free observation until month 12, with monthly monitoring of central memory T-cell frequency (CD45RA^-^CCR7^+^ CD95^+^) and DSA. Advantages: precisely depletes central memory T cells involved in long-term rejection; short-term low-dose cyclophosphamide does not cause severe myelosuppression; dual blockade inhibits both migration and costimulation; theoretically could achieve drug-free tolerance within 6 months. Issues: odulimab has not completed Phase II trials in transplantation; PML risk, though theoretically lower, still warrants vigilance; cyclophosphamide may cause nausea, alopecia, and haemorrhagic cystitis; memory T cells may re-expand, with ~40% becoming DSA-positive within 1 year; only animal data exist, clinical feasibility is very low; requires strict selection of EBV-seropositive recipients to reduce PTLD risk, not suitable for elderly or multimorbid patients. Current evidence level: Preclinical only (36, 47).

## Feasibility analysis and translational barriers

### Multidimensional feasibility assessment

We rated the major protocols from **Table 1** across five domains (cost, operational complexity, risk-benefit ratio, target patient population, and regulatory hurdles) using qualitative High/Medium/Low ratings (**Table 3**).

**Table 3:** Feasibility ratings of major tolerance induction protocols.

Protocol	Cost	Operational complexity	Risk-Benefit ratio	Target population	Regulatory hurdles
T-cell depletion (alemtuzumab/ATG)	Medium	Low	Medium	Low-risk recipients	Low
Costimulation blockade (belatacept ± anti-CD40)	High (belatacept)	Medium	High	Low-medium risk	Medium
Mixed chimerism (MGH/Stanford)	Very High	Very High	High (efficacy) but high toxicity	Highly selected, young, living-donor	Very High
Regulatory cell therapy (Treg/DCreg)	Very High	Very High	Medium	Low-risk, living-donor	Very High
Novel targets (anti-CD45RB, anti-LFA-1, etc.)	High (development)	High	Low (unproven)	Experimental	High

On this basis, costimulation-blockade protocols (belatacept + anti-CD40) offer the best balance of safety, operability, and cost-effectiveness, making them the most deployable in the near term. Mixed chimerism, though most effective, is limited to specialised centres because of its complexity and toxicity.

## Barriers and facilitators for clinical translation

Despite promising results, several barriers hinder widespread adoption: (i) Economic cost – belatacept and anti-CD40 are expensive, and cell therapies require good manufacturing practice (GMP) facilities; (ii) Operational complexity – chimerism and cell therapy demand specialised expertise; (iii) Risk-benefit uncertainty – the risk of late rejection or infection must be weighed against drug toxicity; (iv) Patient selection – robust biomarkers to identify tolerant candidates are lacking; (v) Regulatory – FDA approval for tolerance protocols is stringent, requiring large, long-term trials. Facilitators include improved antibody engineering (e.g., Fc-silent anti-CD40), precision immune monitoring (enzyme-linked immunospot (ELISPOT), Treg/Teff ratio, DSA), and artificial intelligence-assisted decision-making to personalise protocols (65–68). Over the next 5-10 years, the most promising clinically translatable approach remains belatacept/anti-CD40-based CNI-free strategies; the four novel regimens await more robust safety and efficacy data.

## Discussion of organ-specific differences

The evidence presented is predominantly from kidney transplantation. Tolerance biology differs substantially among organs: the liver has inherent immune privilege and higher tolerance rates [~40-50% can withdraw under stringent selection (14)], whereas the lung and heart are more resistant because of constant environmental exposure and high immunogenicity. Pancreas transplantation faces additional challenges from autoimmunity in type 1 diabetes. Thus, sequential protocols must be tailored; for example, liver recipients may need less intensive depletion, whereas lung recipients may require stronger costimulation blockade. We recommend that future trials stratify by organ type.

## Conclusion

Sequential antibody induction strategies have demonstrated feasibility for inducing clinical transplant immune tolerance under rigorous conditions, with mixed chimerism and belatacept sequential protocols being the first to achieve complete immunosuppressant withdrawal in some recipients (18, 25, 46, 63). However, the strategy is still in an early exploratory phase, with a limited beneficiary population; complete tolerance rates vary widely from 10% to 70% across different protocols, and are accompanied by multiple challenges such as increased infection risk, variable suppression of DSA generation, and lack of immune monitoring. Our multidimensional feasibility analysis indicates that costimulation blockade sequential protocols have comprehensive advantages in safety, operability, and cost-effectiveness, representing the most promising pathway for near-term dissemination (46, 69). Chimerism protocols, while most effective, have complexity and risk that confine them to select specialized centers (18, 25). In the future, with integrated advances in antibody engineering (70), precise immune stratification techniques (65), and artificial intelligence-assisted decision-making (66–68), individualized sequential protocols are expected to successfully shift the long-term management model of organ transplantation from “lifelong immunosuppression” to “controlled immune tolerance,” fundamentally improving transplant patients’ quality of life, prognosis, and the global transplant health economic landscape (71–73).
